# A rare case of retroperitoneal extension in Fournier's gangrene: A case report and review of literature

**DOI:** 10.1016/j.amsu.2022.103595

**Published:** 2022-04-09

**Authors:** Sunil Basukala, Yugant Khand, Soumya Pahari, Kunda Bikram Shah, Aashish Shah

**Affiliations:** a Department of Surgery, Shree Birendra Hospital, Chhauni, Kathmandu, 44600, Nepal; b Nepalese Army Institute of Health Sciences – College of Medicine, Sanobharyang, 44600, Kathmandu, Nepal; c Department of Anesthesiology and Critical Care Medicine, Shree Birendra Hospital, Chhauni, Kathmandu, 44600, Nepal

**Keywords:** Fournier's gangrene, Retroperitoneal abscess, Sepsis, Necrotizing fasciitis, Case report

## Abstract

**Introduction and importance:**

Retroperitoneal extension is a rare and fatal complication of Fournier's gangrene (FG) which mandates immediate surgical intervention for better outcome.

**Case presentation:**

A 70-year-old male presented to the emergency department with a history of bilateral painful scrotal swelling for 7 days with fever and abdominal pain for 3 days. On his general examination, he was septic with necrotic patches in the perineum and bilateral scrotum. Imaging revealed soft tissue gas and collections in scrotum extending to the right retroperitoneum with massive collection suggestive of retroperitoneal abscess. Following resuscitation and intravenous antibiotics, immediate exploratory laparotomy was done to drain the retroperitoneal abscess followed by debridement of Fournier's gangrene. The patient remained well on follow up.

**Clinical discussion:**

Fournier gangrene is a fulminant polymicrobial infection of the perineum, scrotum and penis which when complicated by retroperitoneal extension, has a very high mortality. Majority of patients have an immunocompromised condition. Early diagnosis with prompt surgical drainage and debridement (within 6 hours) significantly reduces the mortality.

**Conclusion:**

High index of suspicion, careful clinical examination and timely use of imaging is crucial for early diagnosis of this rare but fatal complication of FG. Furthermore, adequate resuscitation with prompt surgical intervention is the key for a favorable outcome.

## Highlights

• Fournier's gangrene (FG) is a type of necrotizing fasciitis involving the perineum and genital region.
• Retroperitoneal extension of FG is a rare and fatal complication which is usually seen in immunocompromised patients.
• Retroperitoneal abscess should be suspected in patients with FG who present with abdominal pain.

## 1 Introduction

Fournier's gangrene (FG) is a type of necrotizing fasciitis that involves the perineum and genital region [1]. There is a progressive invasion of muscle fascia and the overlying subcutaneous fat in such cases. A higher propensity of fascial involvement in comparison to muscle is noted which is attributed to poorer blood supply in the fascia [2]. The major microbial category for NF is polymicrobial (type I) and monomicrobial (type II) and FG falls into type I category. The major causative microbes include facultative organisms like *Escherichia coli*, *Klebsiella* or *Enterococcus* and also includes anaerobes such as *Bacteroides*, *Fusobacterium*, *Clostridium* [3]. The infection mainly originates from anorectal, genitourinary or cutaneous sources. FG mainly affects the male population but is not uncommon in females [4]. However, it has been noted that there is an increase in mortality amongst female patients having FG due to higher incidence of involvement in retroperitoneal space and abdominal cavity [5]. The clinical presentation of FG evolves within a span of two days to one week and includes features of local swelling, discomfort, crepitus, fever and frank septic shock [6]. This surgical emergency mandates an early debridement which has been associated with better outcomes and survival rate if intervened within 6 hours of diagnosis [7].

There have been very few cases reported of FG with retroperitoneal extension. We present here a rare and potentially fatal case of FG with retroperitoneal extension who was successfully managed with timely surgical debridement of FG and drainage of retroperitoneal abscess. This case report is presented in accordance with the SCARE criteria [8].

## 2 Method

We reported this case following the updated consensus-based Surgical Case Report (SCARE) guidelines [8].

## 3 Case presentation

A 70-year-old retired army serviceman with no known comorbidities, referred from Nepalgunj Military hospital, presented to our emergency department with complaints of pain over the right lower abdomen for the last 3 days and painful bilateral scrotal swelling for 7 days. The abdominal pain was dull aching in nature, initially localized to the right lower abdomen but had progressively radiated to pelvic and bilateral flanks with no known aggravating factors on the day of presentation. There was no history of nausea, vomiting, loose stool or burning urination. His bowel and bladder habits were normal. He was managed in Nepalgunj Military hospital with oral antibiotics and analgesics for painful scrotal swelling. However, his symptoms further worsened followed by pain in abdomen and fever for the past 3 days. His past medical and surgical history, family history, drug and allergic history were insignificant. He is a non smoker and does not consume alcohol.

**Fig. 1:**
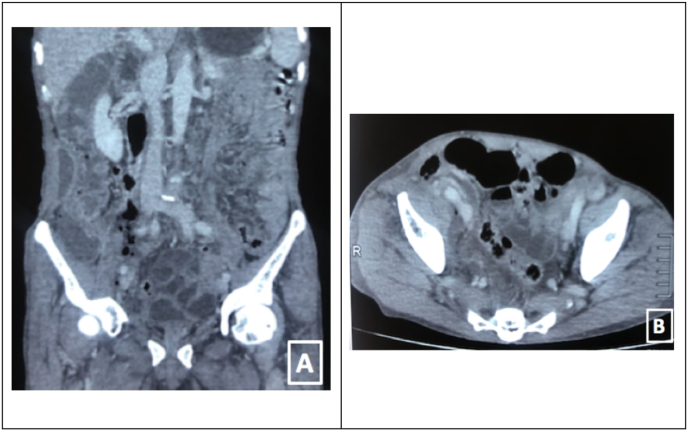
A. Coronal CT image showing gas tracking from the right perirectal area superiorly into the right retroperitoneum to the level of the medial and inferior right kidney. B. Axial CT image showing gas around the right rectum to the sacrum and ascending anterior along the right internal pelvic sidewall.

He was advised for Ultrasonography (USG) abdomen and pelvis which showed right paracolic and paracaecal collection measuring 3 X 3 × 7 cm. With increased intensity of pain in the abdomen, he was further advised for Contrast Enhanced Computed Tomography (CECT) abdomen pelvis to confirm the diagnosis. CECT scan of abdomen and pelvis revealed soft tissue gas in the scrotum and retroperitoneal cavity. It revealed an ill-defined collection measuring 10 X 5 × 4 cm (1040 ml) in size is noted in right abdominopelvic cavity, extending superiorly up to subcostal region with extension to right lumbar, right paracolic gutter and right iliac fossa. Right kidney was displaced anteriorly by the collection suggesting retroperitoneal extension of the collection. Multiple interspersed air foci were noted in the collection extending to the scrotal wall mainly to the right side suggesting the retroperitoneal extension Fig. 1. He was then referred to our hospital for further medical management with a provisional diagnosis of retroperitoneal abscess.

On his arrival to the emergency department, he was febrile (101 °F) and in acute distress due to pain with tachycardia and tachypnea. His vitals were suggestive of septic shock with blood pressure of 80/40 mmHg on presentation. However, after initial fluid resuscitation, his vitals were stabilized. The abdomen was distended and diffusely tender with localized guarding and rigidity on the right lower abdomen. On rectal examination, normal tone, finger stained with stool and no palpable mass were noted. On local examination of the scrotal wall, there was edema with erythema, and patches of blackish discoloration involving both sides of scrotum. The erythema was extending to the right lower abdominal wall. His laboratory investigation revealed an elevated white blood cell count of 21.3 × 10^3^ cells/μL with 62% neutrophils and platelet count of 124 × 10^3^ cells/μL (thrombocytopenia). The biochemical tests showed a creatinine: 1.3 mg%, total bilirubin: 1.5 mg%, direct bilirubin: 0.2 mg%, protein: 5.4 g%, albumin: 2.6 g%, Aspartate Aminotransferase (AST): 128 IU/L, Alanine Aminotransferase (ALT): 127 IU/L, Alkaline phosphatase: 127 IU/L. His Random Blood Sugar (RBS) was 128 mg/dl. The serology was non reactive for HIV, Hepatitis B and Hepatitis C. On radiological examination, X-ray abdomen erect/supine were unremarkable. A provisional diagnosis of retroperitoneal abscess secondary to Fournier's gangrene with sepsis was made and broad spectrum antibiotics were started.

**Fig. 2:**
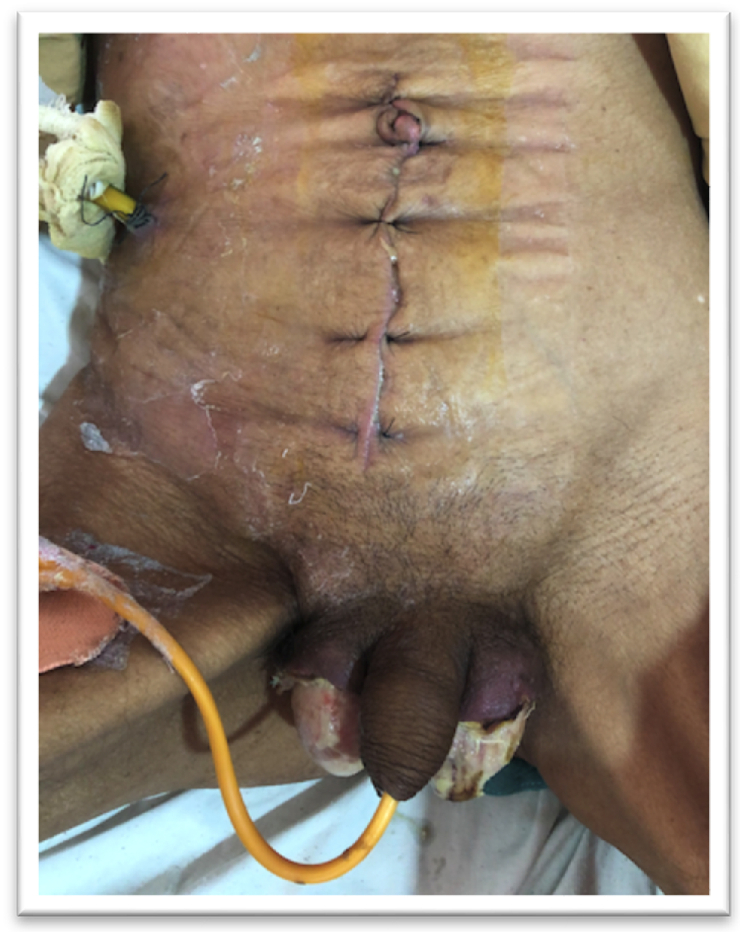
Image of the patient's midline abdominal closure with abdominal drain and debridement of bilateral Fournier's Gangrene.

The patient underwent exploratory laparotomy and debridement of FG on the same day. Intraoperatively, infected necrotic tissues and foul-smelling exudates were found in the right retroperitoneum space requiring a wide excision of the left psoas muscle, lateral abdominal wall, and Gerota's fascia of the right kidney. Approximately 700 ml of purulent collection was drained from right retroperitoneal space and pus was sent for culture and sensitivity. No intraperitoneal collection was noted intraoperatively. A temporary abdominal drain was placed in the right retroperitoneal space and the abdomen was closed after adequate Normal Saline (NS) lavage. This was followed by debridement of Fournier's Gangrene of bilateral scrotum Fig. 2. These findings revealed massive necrotizing fasciitis involving scrotum with sparing of testicles, extending to both inguinal region, right lateral parietal wall, and right psoas muscle. The surgery was performed by an experienced consultant general surgeon in a tertiary care hospital. The postoperative period remained uneventful and he showed improvement in general condition.

## 4 Clinical discussion

Fournier gangrene (FG) is a fulminant polymicrobial infection of the perineum, scrotum and penis. There is progressive obliteration of subcutaneous arteries leading to gangrene of the subcutaneous tissue [6]. The complication of FG includes urinary tract infection, wound infection, renal failure, thromboembolic disease of lower extremities and septic metastasis. There are few case reports of septic metastasis to retroperitoneal space in FG leading to retroperitoneal abscess.

Retroperitoneal abscess has an insidious onset with atypical presentation. The most common source of retroperitoneal abscess is accounted to be genitourinary, gastrointestinal infections, injuries, or malignancy of adjacent retroperitoneal or intraperitoneal organs. Retroperitoneal involvement is seen in less than 30% cases with FG [9]. According to a study by Crepps et al., the majority of patients with retroperitoneal abscess had an immunocompromised condition such as diabetes, malignancy or renal failure [10]. An immunocompromised state also alleviates the occurrence of FG. However, our case had no underlying condition such as diabetes mellitus, tuberculosis or malignancy and presented with scrotal swelling and abdominal pain for one week. The usual presentation in FG varies from erythema, severe scrotal pain and fever. The clinical examination is nonspecific with features of scrotal swelling, hyperemia, discoloration of the skin, putrid smell, crepitus and skin bullae. We believe that the infection metastasized to the retroperitoneal space through inguinal canal or possibly via Colle's fascia. The presentation in our case was however vague leading to a delay in arrival at the hospital. It has been noted that the insidious feature in retroperitoneal abscess leads to diagnostic delay and if immediate drainage is not considered, there will be significant morbidity and mortality [10].

The initial investigation to evaluate FG requires blood workup and imaging. There is an increase in white blood cells with a potential left shift, coagulopathy, hyponatremia and elevated inflammatory markers (C-reactive protein and/or erythrocyte sedimentation rate). The elevation in serum Creatine Kinase and AST levels suggest a deeper infection involving the fascia which also helps differentiate Necrotizing Soft Tissue Infections (NSTI) from cellulitis [11]. The wound culture assists in identifying the microorganism. In case of FG, type I or polymicrobial is more common than monomicrobial infection. Our case had a similar polymicrobial infection reported from serial wound and pus cultures. In addition to laboratory markers, imaging tools also help to gauge the extent of infection. Ultrasound is used to assess edema and thickness of the affected soft tissue. The best initial imaging tool is considered to be Computed Tomography scans considering its high sensitivity and specificity in diagnosing necrotizing fasciitis [12]. We were able to identify a large retroperitoneal collection mainly on the right side of the abdomen with extension to the right inguinoscrotal region. The use of Magnetic Resonance Imaging in diagnosing NSTI can be excluded because it tends to overestimate the extent of deep fascia involvement [13].

In addition to laboratory markers and imaging, various tools have been recognized in aiding the diagnosis of NSTI. The most frequently used tools with high diagnostic value are Laboratory Risk Indicator for Necrotizing Fasciitis (LRINEC) score, NSTI Assessment Score (NAC) and Uludag Fournier's Gangrene Severity Index (UFGSI). The UFGSI is considered to be a powerful indicator in regards to the 1995 Fournier's Gangrene Severity Index scoring system [14,15].

In suspicion of NSTI, a prompt surgical exploration with debridement and drainage along with antibiotic therapy is indicated in high risk patients (such as immunocompromised patients) [16]. This urological emergency mandates empirical antibiotics tailored to gram staining, culture and antibiotic sensitivity tests. The use of antibiotics should be considered up until the patient's hemodynamic condition is stabilized and no further debridement is required. The duration of antibiotic can be tailored for at least two weeks or according to the patient's underlying condition [17]. The use of antibiotics assists to cover retroperitoneal abscess but surgical management is a better approach in management of large retroperitoneal abscess [18]. Due to the large extension of retroperitoneal abscess, immediate exploratory laparotomy and drainage of abscess was performed on our patient. Following surgical debridement, the use of broad spectrum antiseptic solution such as sodium hypochlorite 0.025% (1:20 Dakin solution diluted in sterile water) or povidone iodine is used as initial dressing and considered to have favorable effects on morbidity and mortality [19]. According to a systematic review, the mortality rate in FG reduces significantly if operated within 6 h in comparison to 12 h of presentation [20]. According to observational studies, the mortality rate in FG ranges from 22 to 40% which is still significantly high [21,22]. Though the case of retroperitoneal extension of FG is rare, it is crucial to create awareness of FG especially in today's increasing population of immunocompromised individuals.

## 5 Conclusion

Retroperitoneal involvement with Fournier's Gangrene though rare should be suspected among patients complaining of abdominal pain. Timely diagnosis can be achieved by having a high index of suspicion, proper clinical examination and use of imaging, allowing prompt surgical intervention. For a favorable outcome, adequate resuscitation and IV antibiotics with prompt surgical drainage of retroperitoneal abscess and debridement of FG is crucial.

The primary takeaway lesson from this case report is that the treating physician/surgeon should have a high index of suspicion for retroperitoneal abscess in FG who present with abdominal pain.

## Ethical approval

Not required in our case.

## Sources of funding

None.

## Author contributions

Sunil Basukala (SB) = Conceptualization, Supervision, Sunil Basukala (SB), Yugant Khand (YK), Soumya Pahari (SP) = Writing - original draft, Sunil Basukala (SB), Yugant Khand (YK), Soumya Pahari (SP), Kunda Bikram Shah (KBS), Aashish Shah (AS) = Writing - review & editing, All the authors read and approved the final manuscript.

## Trial registry number

1. Name of the registry: Not applicable
2. Unique Identifying number or registration ID: Not applicable
3. Hyperlink to your specific registration (must be publicly accessible and will be checked): Not applicable

## Guarantor

Sunil Basukala (SB), MD in Hospital Administration (MDHA), Master of Surgery (MS), Assistant Professor, Shree Birendra Hospital, Nepalese Army Institute of Health Sciences (NAIHS), 44600, Nepal. Email: sunil.basukala@naihs.edu.np.

## Consent

Written informed consent was obtained from the patient for publication of this case report and accompanying images. A copy of the written consent is available for review by the Editor-in-Chief of this journal on request.

## Provenance and peer review

Not commissioned, externally peer-reviewed.

## Declaration of competing interest

All authors declare that they have no conflict of interest.
